# New Morbidity and Comorbidity Scores based on the Structure of the ICD-10

**DOI:** 10.1371/journal.pone.0143365

**Published:** 2015-12-14

**Authors:** Jürgen Stausberg, Stefan Hagn

**Affiliations:** 1 Institute for Medical Informatics, Biometry and Epidemiology, Faculty of Medicine, University Duisburg-Essen, Essen, Germany; 2 Zuckerbacherweg 6, 84036 Landshut, Germany; ISPO, ITALY

## Abstract

Measures of morbidity and comorbidity are frequently used for the control of confounding, particularly in health services research. Several proposals for those measures are defined with ICD-coded diagnoses available in hospital routine data. However, a measure that makes use of the ICD structure is missing. Objective of this work was to elaborate the power of the ICD structure for defining morbidity and comorbidity measures. Routine data from three German hospitals with inpatients discharged 2008 were used for model development; routine data from 36 German hospitals with inpatients admitted and discharged 2010 were used for model evaluation. Two different risk models were developed, one based on ICD-10 chapters, the other based on ICD-10 groups. The models were transformed into sum scores using whole-number weights. Models and scores were compared with the Charlson Index and the Elixhauser Comorbidities using the receiver operating characteristic. Dependent variable was hospital death. Logistic regression was used to derive the new models. Charlson Index and Elixhauser Comorbidities were mapped to the German ICD-10. According to the receiver operating characteristic, the quality of the measures based on the structure of the ICD-10 was superior compared with the Charlson Index and the Elixhauser Comorbidities. The best result was achieved with the measure based on ICD-10-groups with an area under curve of 0.910 (95% confidence interval = 0.907–0.913). The sum scores showed a comparable performance. The developed new measures may be used to control for confounding.

## Introduction

Measures of morbidity and comorbidity are used, when the overall burden of diseases is regarded as a confounder. This is particularly relevant in health services research, in quality management, in pay-for-performance approaches, and in health economics. Four reasons had been mentioned for measuring morbidity [1]:

- control of confounding,
- identification of effect modifiers,
- prediction of outcome, and
- improvement of statistical efficiency.

Morbidity can be defined several fold [2]:

1. absence of physical and mental well-being,
2. a sick individual,
3. diseases of a sick individual, or
4. the duration of a disease.

For this work, definition three is applied. If morbidity is the set of diseases of an individual, comorbidity might be different. Firstly, comorbidity can be regarded as diseases that exist additionally to one or several diseases of interest [1, 3]. In routine data of inpatient care, the principle diagnosis is frequently taken as the disease of interest, the set of secondary diagnoses as comorbidity. Secondly, comorbidity could be defined as the coexistence of several diseases, without the necessity to exclude a disease of interest [4]. Thirdly, complications could be separated from preexisting diseases and those summarized as comorbidity [5, 6]. All three approaches for the definition had been applied in works about comorbidity measures.

The calculation of morbidity and comorbidity measures from diagnoses coded with the International Statistical Classification of Diseases and Related Health Problems (ICD) has reached great interest (cf. Birkmeyer et al. [7] as application example and Quan et al. [8] as methodological work). In most of the developed countries that reimburse inpatient cases with diagnosis related groups (DRGs), routine data can be used without the necessity of additional data acquisition at least for inpatient care. Diagnoses coded with the ICD are part of the routine data. Several proposals for the definition of morbidity or comorbidity measures based on ICD-coded diagnoses had been made [8–12]. These definitions make use of two different comorbidity measures, the Charlson Index published in 1987 [13] and the Elixhauser Comorbidities published in 1998 [10]. Both measures and their derivatives were developed through a combination of expert opinions and statistical analyses. Meanwhile, several reviews are available comparing the performance of the different measures in different settings [14, 15]. Yurkovich et al. showed that there is room for improvement [15]. Predicting hospital mortality, analyses based on the Charlson Index received very heterogeneous results with an area under curve (AUC) between 0.608 and 0.860, scores based on the Elixhauser Comorbidities with an AUC between 0.632 and 0.878.

As far as the author knows, the feasibility of the native ICD structure as basis of morbidity or comorbidity measures has not been analyzed yet. Therefore, this analysis intended to make use of the ICD structure for defining such measures, not only to code expert opinions about those measures with the ICD as in previous work. Furthermore, the quality of respective measures was compared with ICD-based definitions of the Charlson Index and the Elixhauser Comorbidities. In-hospital death was used as the dependent variable. The study was split up in the development and the validation of the measures using different convenient samples of inpatient cases.

## Materials and Methods

### Data sets

Anonymized routine data of 51,898 inpatient cases discharged in 2008 from three acute-care hospitals were used for the development of the model (cf. Table 1). The hospitals belonged to one hospital group in a large city of Germany. Newborns were excluded from the analysis. Hospital mortality was 2.4%; the mean number of secondary diagnoses was 3.55 with a standard deviation of 3.33. For evaluation, anonymized routine data of 435,076 inpatient cases admitted and discharged in 2010 from 36 acute-care hospitals in Germany were included. Those hospitals participated in a nationwide quality benchmarking project of ecclesiastical institutions. Hospital mortality was 2.3%; the mean number of secondary diagnoses was 4.61 with a standard deviation of 4.56. All diagnoses, i.e. principal as well as secondary ones were available, coded with the ICD-10 German Modification 2008 (ICD-10-GM 2008) or ICD-10-GM 2010. A present on admission flag was not available in both data sets. It is still missing in hospital routine data in Germany. Furthermore, a patient identifier was missing too. Therefore, it was not able to assign different inpatient cases to an individual.

**Table 1:** Description of the population.

	Development data set	Evaluation data set
Hospitals (number)	3	36
Inpatients (number)	51,898	435,076
Year of discharge	2008	2010
Hospital mortality (percent)	2.4%	2.3%
Length of stay in days (mean ± standard deviation)	6.65 ± 8.44	6.35 ± 7.24
Age in years (mean ± standard deviation)	55.7 ± 23.3	55.3 ± 25.1
Female (percent)	54.3%	54.1%
Number of secondary diagnoses per inpatient (mean ± standard deviation)	3.55 ± 3.33	4.61 ± 4.56

In alphabetical order, the five most frequent principal diagnoses in the develeopment data set were atherosclerosis of arteries of extremities Fontaine stage IIA (ICD-10-GM code I70.21), left ventricular failure with severe limitations (I50.14), obstructive sleep apnoea syndrome (G47.31), other forms of angina pectoris (I20.8), and overlapping lesion of bronchus and lung (C34.8). The five most frequent principal diagnoses in the evaluation data set were concussion (S06.0), left ventricular failure with severe limitations (I50.14), other primary gonarthrosis (M17.1), singleton, born in hospital (Z38.0), and syncope and collapse (R55).

### Model and score development

For the new measures concerning the ICD-10-structure, two risk models were developed using logistic regression with hospital death as dependent and the ICD-10-chapters or ICD-10-groups as independent variables. In the final models, non-significant chapters or groups were excluded. Both models were developed using the principal diagnosis (PDX) as well as secondary ones (SDX). For each patient, the set of secondary diagnoses was trimmed as follows. Additional codes were excluded. On the one hand additional codes were identified by the dagger and asterisk-system that is used in the ICD-10 internationally. Typically, dagger codes describe the etiology and asterisk codes the localization of a disease. The use of dagger codes is obligatory, the additional use of asterisk codes optional. On the other hand additional codes are flagged by an exclamation mark in the ICD-10-GM nationally. Those codes are not part of the international version of the ICD-10. A disease occurring left and right simultaneously (e.g. both right hip and left hip arthrosis) was counted only once. Furthermore, a code recorded several times was not counted but once.

The coefficients of the models were transformed into a score according to Perneger et al. [16] and Sullivan et al. [17]. The regression coefficient with the minimal absolute value received the weight “1” or “-1” depending on its prefix. The other regression coefficients were transformed by dividing their absolute value by the minimal absolute value rounded to a whole number preserving the prefix. The score of an individual case is then calculated as the sum of the weights counting a group or chapter only once.

Other variables as sex and gender were not included into the models, even if they influence hospital mortality. The goal was not to find the best model explaining hospital mortality applying the whole range of patient characteristics. This is typically the case in risk models used to calculate a hospital standardized mortality ratio (HSMR) [18, 19]. Those risk models include a comorbidity score as one out of several independent variables. It would be contradictory if the comorbidity score itself includes other variables.

### Measures for comparison

Charlson et al. calculated the mortality risk of specific diseases using univariate statistical procedures [13]. An exclusion of a disease of interest is not reported. A relevant risk was identified for 19 conditions. A weight of 1, 2, 3 or 6 was given to each condition. The Charlson Index is the sum of the weights. In this analysis, a reduced version with 17 conditions with the original weights of Charlson et al. was used [9]. The definition of the conditions with the ICD 10th Revision Canadian version (ICD-10-CA) by Quan et al. [8] was mapped to the ICD-10-GM 2010.

Elixhauser et al. published a list of 30 diseases coded with the ICD 9th Revision Clinical Modifications (ICD-9-CM) that showed a risk for length of stay, costs, and mortality [10]. The principal diagnosis was excluded. Later on, van Walraven et al. [20] added a scoring algorithm based on 21 out of the 30 Elixhauser Comorbidities with weights between -7 to 12. The occurrence of negative weights is an artifact resulting from the statistical methodology. A negative weight does not denote diseases preventing patients from dying during hospital stay. The score is then calculated as the sum of the weights. The definition of the comorbidities with the ICD-10-CA by Quan et al. was also mapped to the ICD-10-GM 2010.

For evaluation, each case was characterized by values of six different models: Charlson Index, score of Elixhauser Comorbidities, risk according to the model based on ICD-10-chapters, risk according to the model based on ICD-10-groups, ICD-10-chapter-score, and ICD-10-group-score. All six models were applied to the data once excluding and once including the PDX leading to 12 different measures.

### Statistics and software

The accordance between the measures was checked using the non-parametric correlation coefficient of Spearman. The methodological quality of the measures was analyzed calculating the AUC of the receiver operating characteristic (ROC) with hospital death as the dependent variable. Significance was assumed with a p < = 0.05 (both sides). Goodness of fit was analyzed with Nagelkerke’s R^2^. The data were maintained with the relational database management system Microsoft Access. The correlation analysis and the logistic regressions were done with IBM SPSS Statistics. All ICD-10-chapters were considered in a first regression analysis (method ENTER). Non-significant chapters were excluded from the final model in a second regression analysis. The logistic regression with ICD-10-groups was run forward stepwise (method FSTEP). Therefore, only significant variables remained in the model.

### Ethics statement

The data were provided for this analysis completely anonymized. Therefore, an approval of an ethics committee or another respective institution was not required. Because of anonymization, a written consent was not required as well.

## Results

### Measures based on the ICD structure

The first measure using the ICD structure was defined on the level of chapters. The ICD-10-GM is divided into 22 chapters. Five chapters were excluded:

- Chapter I “Certain infectious and parasitic diseases“, because it includes many additional codes.
- Chapter XV “Pregnancy, childbirth and the puerperium“, because it does not describe diseases primarily.
- Chapter XX “External causes of morbidity and mortality”, because it includes solely additional codes.
- Chapter XXI “Factors influencing health status and contact with health services”, because the conditions that are classified here are usually not regarded as a disease.
- Chapter XXII “Codes for special purposes”, because it includes solely undefined codes.

The second measure using the ICD structure was defined on the level of groups. In some parts of the ICD-10, groups are further structured hierarchically. In those parts, only the uppermost group was considered:

- C00-C97 Malignant neoplasms
- M00-M25 Arthropathies
- M40-M54 Dorsopathies
- M60-M79 Soft tissue disorders
- M80-M94 Osteopathies and chondropathies

Groups belonging to the chapters I, XV, XX, XXI, and XXII (see above) were excluded as well. In the calculation of both measures, codes including death as part of their definition were excluded:

- Codes within group R95-R99 Ill-defined and unknown causes of mortality
- I46.1 Sudden cardiac death, so described
- O95 Obstetric death of unspecified cause (excluded due to the chapter selection as well)
- O96 Death from any obstetric cause occurring more than 42 days but less than one year after delivery (excluded due to the chapter selection as well)
- P95 Fetal death of unspecified cause

From 241 ICD-10-GM-groups, 165 remained for the measure based on ICD-10-groups.

The final models included 13 ICD-10-GM-chapters (S1 Appendix) and 42 ICD-10-GM-groups (S2 Appendix). The model based on ICD-10-chapters received an AUC = 0.863 (confidence interval (CI) = 0.853–0.872), the model based on ICD-10-groups an AUC = 0.916 (CI = 0.909–0.923). The ICD-10-chapter-score included weights between -6 and +7 with a maximal sum of 32; the ICD-10-group-score included weights between -131 and +6 with a maximal sum of 71.

Nagelkerke’s R^2^ was 0.229 for the model based on ICD-10-chapters and 0.330 for the model based on ICD-10-GM-groups.

### Evaluation

Tables 2 and 3 show the results of the correlation analysis. All bivariate comparisons were statistical significant with p< = 0.01. The maximum of the correlation coefficient for different models was 0.734 between the Charlson Index and the Elixhauser Comorbidities including the PDX and 0.711 excluding the PDX. The minimum was 0.488 between the ICD-10-group-score and the Charlson Index including the PDX and 0.418 excluding the PDX. The correlation coefficients were equal and higher than 0.990 for the comparisons of the scores with the risks recalculated using the original ß-coefficients. Having correlation coefficients between 0.451 and 0.539, the new measures are less concordant among each other than the Charlson Index and the Elixhauser comorbidities.

**Table 2:** Non-parametric correlation coefficient from the correlation analysis of the comorbidity measures including the PDX.

Measure	1	3	5	7	9	11
Charlson PDX+SDX (1)	1.000	0.734	0.542	0.502	0.550	0.488
Elixhauser PDX+SDX (3)		1.000	0.535	0.594	0.539	0.589
ICD-10 chapters PDX+SDX (5)			1.000	0.539	0.999	0.517
ICD-10 groups PDX+SDX (7)				1.000	0.537	0.993
ICD-10-chapter-score PDX+SDX (9)					1.000	0.514
ICD-10-group-score PDX+SDX (11)						1.000

PDX = Principal diagnosis, SDX = Secondary diagnoses. All results are statistical significant with p< = 0.01.

**Table 3:** Non-parametric correlation coefficient from the correlation analysis of the comorbidity measures excluding the PDX.

Measure	2	4	6	8	10	12
Charlson SDX (2)	1.000	0.711	0.556	0.434	0.561	0.418
Elixhauser SDX (4)		1.000	0.544	0.555	0.547	0.548
ICD-10 chapters SDX (6)			1.000	0.480	0.999	0.451
ICD-10 groups SDX (8)				1.000	0.479	0.990
ICD-10-chapter-score SDX (10)					1.000	0.449
ICD-10-group-score SDX (12)						1.000

PDX = Principal diagnosis, SDX = Secondary diagnoses. All results are statistical significant with p< = 0.01.

Table 4 shows the results of the ROC-analysis. All results were different from the null hypotheses (AUC = 0.5) with p<0.001. The initial performance of the new models was confirmed with an AUC = 0.856 (CI = 0.852–0.859) for the model based on ICD-10-chapters and an AUC = 0.910 (CI = 0.907–0.913) for the model based on ICD-10-groups. According to their 95%-confidence limits, the scores performed equally to the raw models. Inclusion of the PDX did not change the performance of the model based on ICD-10-chapters; the model based on ICD-10-groups showed a slightly higher AUC = 0.910 (CI = 0.907–0.913) with PDX than without PDX (AUC = 0.894, CI = 0.891–0.897). To check for a bias due to the extreme negative weight of group K35-K39 “Diseases of appendix” the scores based on ICD-10-groups were additionally analyzed excluding diagnoses codes of that group (results not shown in Table 4). The results were identical to the original scores with an AUC = 0.909 (CI = 0.906–0.911) with PDX and AUC = 0.891 (CI = 0.888–0.895) without PDX.

**Table 4:** Area under curve (AUC) from the ROC-analysis.

Measure	Diagnoses	AUC	Asymptotic 95%-confidence interval	
			lower limit	upper limit
Charlson	SDX	0.764	0.759	0.769
	PDX+SDX	0.792	0.788	0.797
Elixhauser	SDX	0.815	0.810	0.819
	PDX+SDX	0.829	0.825	0.833
ICD-10 chapters	SDX	0.853	0.850	0.857
	PDX+SDX	0.856	0.852	0.859
ICD-10 groups	SDX	0.894	0.891	0.897
	PDX+SDX	0.910	0.907	0.913
ICD-10-chapter-core	SDX	0.854	0.850	0.857
	PDX+SDX	0.856	0.852	0.859
ICD-10-group-score	SDX	0.890	0.887	0.894
	PDX+SDX	0.908	0.905	0.911

PDX = Principal diagnosis, SDX = Secondary diagnoses

Including the PDX, the Charlson Index received an AUC = 0.792 (CI = 0.788–0.797) and the Elixhauser Comorbidities an AUC = 0.829 (CI = 0.825–0.833), being significantly lower than all results achieved with the new models based on the ICD structure.

## Discussion

Two new risk models for morbidity and comorbidity based on the ICD structure were developed using logistic regression analyses with hospital death as dependent variable. One of them was based on 13 out of 22 chapters of the ICD-10-GM, the other one on 42 out of 241 groups. The performance results of the development stage were confirmed in the evaluation using a totally independent data set of more than 400,000 inpatient cases. Moreover, the sum score for both models showed a comparable performance to the application of the raw model. This is important to provide an easy-to-use-approach for the application of the models in future projects. Depending on the use case, the scores are applicable with and without the PDX. Including the PDX will address morbidity, excluding the PDX will address comorbidity. The weight determined for ICD-10-group K35-K39 “Diseases of appendix” appears as an artifact. Nevertheless, diagnoses from that group could be excluded without lowering the score’s performance.

The concordance of the new measures among each other and with the Charlson Index and the Elixhauser Comorbidities was weaker than the concordance between the Charlson Index and the Elixhauser Comorbidities. Charlson Index and Elixhauser Comorbidities might share a common tradition that is not addressed by the new measures. Surprisingly, the concordance between the ICD-based measures was lower than the concordance between their competitors. The formal hierarchy of the ICD-10 might not correctly reflect the clinical relationship between diseases. As a consequence, the meaning of a branch in terms of morbidity or comorbidity changes between chapters and groups.

In the ROC-analysis, the measure based on ICD-10-groups showed the best performance, independently of the exclusion or inclusion of the PDX. As far as the authors know, an AUC of 0.910 (CI 0.907–0.913) for hospital death is the best value published for ICD-based measures ever, if the measure is applied without co-variables like sex and age. A superior result was published by Quail et al. combining the Elixhauser Comorbidities with a base model [21]. They calculated an AUC of 0.913 (CI 0.910–0.916) for death in the year following the base year in a general population cohort of 662,423 people in Canada.

The superiority of the Elixhauser Comorbidities to the Charlson Index was recognized in several studies before. E. g. Southern et al. [22] received an AUC of 0.704 for the Charlson Index in the adaptation of Deyo et al. and an AUC of 0.793 for the Elixhauser Comorbidities. In that study, the principal and the secondary diagnoses coded with the ICD-9-CM had been included. Ou compared both comorbidity measures defined with the ICD-10 [23]. The Charlson Index received an AUC of 0.860 in the prediction of hospital mortality, the Elixhauser Comorbidities an AUC of 0.870, including all diagnoses for both measures. In the work of Chu et al. [24], results between 0.708 and 0.737 were reached for both measures in the prediction of hospital mortality for acute myocardial infarction and chronic obstructive pulmonary disease. The results derived from the German dataset for the international measures are similar. This might be an indicator of the high quality of diagnoses documentation for inpatient care in Germany [25]. Together with the USA and Belgium, the mean number of secondary diagnoses in German hospitals is the highest in 14 member states of the Organisation for Economic Co-operation and Development (OECD), all using some kind of a DRG-like reimbursement system [26]. Therefore, a general underreporting of diagnoses could be ruled out. However, reimbursement incentives could induce an improper up-coding [27]. As far as we know, that did not happen in Germany due to a strong quality control of the medical documentation on behalf of the statutory health insurances [28, 29].

The risk models based on the ICD structure reached reasonable results. The model applying the chapters of the ICD-10 achieved results that are reasonably higher than those of the Charlson Index and the Elixhauser Comorbidities. A further differentiation of the chapters to the groups of the ICD-10 then improved the model’s quality yet again. Obviously, ICD-10-groups cover comorbidity relevant for hospital mortality quite better than the chapters. A further automatic differentiation is difficult due to the high number of about 14,000 terminal codes in the ICD-10-GM, a number too large as to include them as independent variables into the analysis. The results presented in this paper demonstrate the promising approach of using the native structure of the ICD-10 as basis for morbidity and comorbidity measures.

The presented scores might not be appropriate for every setting. Studies focusing on homogeneous groups of patients suffering from an index disease might benefit from a disease specific score. Furthermore, the presented scores are justified by hospital mortality as proxy for morbidity. If other proxies are favored, scores developed specifically with those proxies as dependent variable might be better.

### Study limitations

The presented study made use of diagnoses recorded and coded in hospitals routine data to estimate the level of morbidity and comorbidity of inpatients. An evaluation of other sources—for example a subjective assessment of the patients’ comorbidity level by experts—was not part of the study. Furthermore, there seems to be a mismatch in some ICD-10 groups and chapters between the weights and the perceived clinical relevance. Those ICD-10 groups and chapters might indicate situations going beyond the specific diseases. Further research could elaborate options to keep the presented approach based on the structure of the ICD-10 while adapting the model to the clinical appraisal.

One might claim that the international approaches perform inferior in Germany due to differences in case mix, etc. This concern can be rejected because the results achieved in German data are equal to the results published in the literature.

Morbidity and comorbidity as well as the respective measures are complex constructs that could be operationalized in several ways, for example:

- diagnoses could be coded differently with the same classification (see the differences between [9] and [12]),
- conditions can be included or excluded (see the smaller version of the Charlson Index proposed by Halfon et al. [11]),
- different weight scores could be used (see [30] for alternative weights for the Charlson Index),
- the principal diagnosis can be included or excluded,
- complications could be considered separately (as in [31]) and
- the dependent variable could be changed.

Complications were not excluded from the models and the data. At least the exclusion of complications from model development is sometimes mentioned as an important feature. However, there is first evidence, that not the frequency of complications determines hospital mortality but rather the handling of complications [32]. If this is true, the exclusion of complications in risk adjustment could lead to wrong conclusions, if a score is applied in research concerned with mortality as adverse event. Therefore, further research should focus on the methodological role of those characteristics for measuring morbidity and comorbidity. Furthermore, the applicability of the scores in other research settings like outpatient care and its performance regarding prospective mortality (e. g. one year) should be analyzed in subsequent studies.

The use of routine data that are primarily recorded for reimbursement purposes might be regarded as problematic due to several reasons. Diagnoses are not necessarily clinically verified, routine data might exaggerate the disease burden due to an inadequate up-coding, routine data might be incomplete because information is missing that is financially not relevant, and diagnoses might be imprecise due to the coding by administrative staff.

## Supporting Information

S1 Appendix Risk model based on ICD-10-chapters. Table A shows the variables along with their coefficients and weights of the risk model based on ICD-10-chapters.
(DOCX)

S2 Appendix Risk model based on ICD-10-groups. Table B shows the variables along with their coefficients and weights of the risk model based on ICD-10-groups.
(DOCX)
