# Supplementary material for: New Morbidity and Comorbidity Scores based on the Structure of the ICD-10
Source: PLoS One. 2015 Dec 14;10(12):e0143365. doi: 10.1371/journal.pone.0143365 (PMC4677989; doi:10.1371/journal.pone.0143365)
Supplement: S1 Appendix — Table A shows the variables along with their coefficients and weights of the risk model based on ICD-10-chapters. (DOCX) [file pone.0143365.s001.docx]

**S1 Appendix. Risk model based on ICD-10-chapters.** Table A shows the variables along with their coefficients and weights of the risk model based on ICD-10-chapters.

**S1 Table A.**

| ICD-10-chapter | | Coefficient β | Weight | Inpatients |
| --- | --- | --- | --- | --- |
| Code | Title |  |  | Number^#^ |
| II | Neoplasms | 1.049336658 | 5 | 58,140 |
| III | Diseases of the blood and blood-forming organs and certain disorders involving the immune mechanism | 0.459510071 | 2 | 44,534 |
| V | Mental and behavioural disorders | -0.442068895 | -2 | 64,041 |
| VII | Diseases of the eye and adnexa | -0.877651609 | -4 | 11,926 |
| VIII | Diseases of the ear and mastoid process | -0.67094637 | -3 | 9,500 |
| IX | Diseases of the circulatory system | 0.640448388 | 3 | 211,473 |
| X | Diseases of the respiratory system | 1.057717041 | 5 | 76,526 |
| XII | Diseases of the skin and subcutaneous tissue | 1.299740037 | 6 | 22,123 |
| XIII | Diseases of the musculoskeletal system and connective tissue | -0.981346104 | -4 | 70,583 |
| XIV | Diseases of the genitourinary system | 0.879224801 | 4 | 88,533 |
| XVII | Congenital malformations, deformations and chromosomal abnormalities | -1.247307158 | -6 | 6,301 |
| XVIII | Symptoms, signs and abnormal clinical and laboratory findings, not elsewhere classified | 1.513604162 | 7 | 128,796 |
| XIX | Injury, poisoning and certain other consequences of external causes | -0.219350445 | -1 | 77,641 |
| Intercept |  | -5.540391471 |  |  |

^#^Number of inpatients in the evaluation data set with at least one code from the ICD-10-chapter as principal or as secondary diagnosis. The total nummer of inpatients was 435,076.
