# Supplementary material for: New Morbidity and Comorbidity Scores based on the Structure of the ICD-10
Source: PLoS One. 2015 Dec 14;10(12):e0143365. doi: 10.1371/journal.pone.0143365 (PMC4677989; doi:10.1371/journal.pone.0143365)
Supplement: S2 Appendix — Table B shows the variables along with their coefficients and weights of the risk model based on ICD-10-groups. (DOCX) [file pone.0143365.s002.docx]

**S2 Appendix. Risk model based on ICD-10-groups.** Table B shows the variables along with their coefficients and weights of the risk model based on ICD-10-groups.

**S2 Table B.**

| ICD-10-group | | Coefficient β | Weight | Inpatients |
| --- | --- | --- | --- | --- |
| Code | Title |  |  | Number^#^ |
| C00-C97 | Malignant neoplasms | 1.382322174 | 4 | 42,623 |
| D10-D36 | Benign neoplasms | -1.729370052 | -5 | 11,125 |
| D60-D64 | Aplastic and other anaemias | 0.360608328 | 1 | 27,400 |
| D65-D69 | Coagulation defects, purpura and other haemorrhagic conditions | 0.406890185 | 1 | 11,725 |
| E00-E07 | Disorders of thyroid gland | -0.624463437 | -2 | 35,255 |
| E50-E64 | Other nutritional deficiencies | -2.330218598 | -7 | 2,927 |
| E65-E68 | Obesity and other hyperalimentation | -0.694485048 | -2 | 20,070 |
| F10-F19 | Mental and behavioural disorders due to psychoactive substance use | -0.948333075 | -3 | 17,105 |
| F30-F39 | Mood [affective] disorders | -1.01474928 | -3 | 16,655 |
| G10-G14 | Systemic atrophies primarily affecting the central nervous system | 1.629455981 | 5 | 369 |
| G90-G99 | Other disorders of the nervous system | 1.105892816 | 3 | 3,428 |
| H53-H54 | Visual disturbances and blindness | -1.459865834 | -4 | 2,887 |
| I10-I15 | Hypertensive diseases | -0.547487145 | -2 | 164,888 |
| I26-I28 | Pulmonary heart disease and diseases of pulmonary circulation | 0.586777751 | 2 | 5,510 |
| I30-I52 | Other forms of heart disease | 0.954756847 | 3 | 79,932 |
| I60-I69 | Cerebrovascular diseases | 0.784098054 | 2 | 23,570 |
| I70-I79 | Diseases of arteries, arterioles and capillaries | 0.392941297 | 1 | 20,673 |
| I80-I89 | Diseases of veins, lymphatic vessels and lymph nodes, not elsewhere classified | -0.498530748 | -1 | 14,802 |
| J09-J18 | Influenza and pneumonia | 1.019041308 | 3 | 14,773 |
| J30-J39 | Other diseases of upper respiratory tract | -1.226031753 | -3 | 11,621 |
| J60-J70 | Lung diseases due to external agents | 1.615969507 | 5 | 2,544 |
| J80-J84 | Other respiratory diseases principally affecting the interstitium | 1.150641776 | 3 | 1,258 |
| J90-J94 | Other diseases of pleura | 0.59419548 | 2 | 10,150 |
| J95-J99 | Other diseases of the respiratory system | 1.186743773 | 3 | 21,361 |
| K35-K38 | Diseases of appendix | -46.66377343 | -131 | 3,350 |
| K40-K46 | Hernia | -0.801183181 | -2 | 13,400 |
| K55-K63 | Other diseases of intestines | 0.439516451 | 1 | 34,859 |
| K65-K67 | Diseases of peritoneum | 0.979266753 | 3 | 5,724 |
| K70-K77 | Diseases of liver | 0.65651189 | 2 | 8,997 |
| L80-L99 | Other disorders of the skin and subcutaneous tissue | 1.234806235 | 3 | 11,906 |
| M00-M25 | Arthropathies | -0.847906446 | -2 | 30,132 |
| M40-M54 | Dorsopathies | -0.951716303 | -3 | 24,767 |
| N17-N19 | Renal failure | 1.311218113 | 4 | 39,206 |
| N25-N29 | Other disorders of kidney and ureter | -1.301579848 | -4 | 2,619 |
| N30-N39 | Other diseases of urinary system | -0.35952431 | -1 | 30,850 |
| Q35-Q37 | Cleft lip and cleft palate | 2.17741583 | 6 | 51 |
| R10-R19 | Symptoms and signs involving the digestive system and abdomen | 0.740957219 | 2 | 48,049 |
| R30-R39 | Symptoms and signs involving the urinary system | 0.357567453 | 1 | 26,612 |
| R40-R46 | Symptoms and signs involving cognition, perception, emotional state and behaviour | 0.855376719 | 2 | 14,707 |
| R50-R69 | General symptoms and signs | 1.184741694 | 3 | 46,312 |
| T15-T19 | Effects of foreign body entering through natural orifice | 1.972671303 | 6 | 667 |
| T80-T88 | Complications of surgical and medical care, not elsewhere classified | -0.492815662 | -1 | 25,965 |
| Intercept | | -5.137019062 |  |  |

^#^Number of inpatients in the evaluation data set with at least one code from the ICD-10-group as principal or as secondary diagnosis. The total nummer of inpatients was 435,076.
